# Albuminuria, cardiovascular risk factors and disease management in subjects with type 2 diabetes: a cross sectional study

**DOI:** 10.1186/1472-6963-8-226

**Published:** 2008-11-05

**Authors:** Christa Meisinger, Margit Heier, Rüdiger Landgraf, Michael Happich, H-Erich Wichmann, Wolfgang Piehlmeier

**Affiliations:** 1 Helmholtz Zentrum München, German Research Center for Environmental Health (GmbH), Institute of Epidemiology, Neuherberg, Germany; 2 Central Hospital of Augsburg, MONICA/KORA Myocardial Infarction Registry, Augsburg, Germany; 3 University of Munich, Department of Internal Medicine, Diabetes Centre, Germany; 4 Eli Lilly and Company, Bad Homburg, Germany; 5 IBE, Chair of Epidemiology, University of Munich, Germany

## Abstract

**Background:**

Epidemiological studies have shown that microalbuminuria is an important risk factor for arteriosclerosis, coronary heart disease and other vascular diseases in persons with type 2 diabetes. In the present study we examined the prevalence and risk factors for micro- and macroalbuminuria and examined glycemic control as well as treatment of modifiable cardiovascular risk factors in persons with known type 2 diabetes in Germany.

**Methods:**

The presented data were derived from the 'KORA Augsburg Diabetes Family Study', conducted between October 2001 and September 2002. Participants were adults aged 29 years and older with previously diagnosed type 2 diabetes (n = 581). Microalbuminuria was defined as an albumin-creatinine ratio of 30 to 300 mg/g, and macroalbuminuria as an albumin-creatinine ratio of more than 300 mg/g.

**Results:**

Microalbuminuria was revealed in 27.2% and macroalbuminuria in 9.0% of the 581 included diabetic persons. Multivariable regression analysis identified HBA1c, duration of diabetes, systolic blood pressure, serum creatinine, smoking and waist circumference as independent risk factors associated with albuminuria (micro- or macroalbuminuria). Relatively few persons with type 2 diabetes achieved treatment targets of HbA1c < 7% (46.6%), total cholesterol < 200 mg/dl (44.1%), and LDL cholesterol < 100 mg/dl (16.0%). Optimal HDL cholesterol values (> 45 mg/dl in men, > 55 mg/dl in women) were found in 55.8%, and blood pressure values < 130 and < 85 mmHg in 31.3% of the persons

**Conclusion:**

Albuminuria is common among German persons with known type 2 diabetes. Despite evidence-based guidelines, only a small proportion of type 2 diabetic persons achieved the recommended levels of glycemic control and control of cardiovascular risk factors.

## Background

Throughout the world, the number of people developing type 2 diabetes mellitus is increasing dramatically. At the present time the disease affecting around 171 million people worldwide and the World Health Organization predicts that this number will rise to 366 million by 2030 [1]. Consequently, the number of people developing diabetes-related complications will increase. Microalbuminuria is a common complication of diabetes and appears to be a strong predictor of subsequent development of overt diabetic nephropathy [2], which is the leading cause of end-stage renal disease in the western world [3,4]. Without any intervention, among type 2 diabetic patients about 20–40% with microalbuminuria progress to overt nephropathy and finally approximately 20% develop end stage renal failure [5,6]. However, microalbuminuria does not only predict future risk of renal injury but is also considered to be associated with an increased risk of cardiovascular events and mortality [7].

Prior studies have shown that the risk factors correlated with the progression of nephropathy in persons with type 2 diabetes are blood pressure, lipid levels, obesity, cigarette smoking, glycaemic control, and anemia [8,9]. Thus, studies on diabetes related complications are very important to estimate the consequence and burden of diabetes. The aims of the present study are to determine the prevalence of and factors associated with micro- and macroalbuminuria in persons with type 2 diabetes and to examine the achievement of international guideline targets with regard to glycaemic control and the management of modifiable cardiovascular risk factors.

## Methods

### Study population

The presented data were derived from the 'Augsburg Diabetes Family Study' which was conducted between October 2001 and September 2002. The primary goal of the study was to enrol families to investigate the role of genes as well as environmental factors in the development of type 2 diabetes [10]. Families were ascertained through an index proband with known type 2 diabetes having at least one full sib or both parents willing to participate in the study. All index probands came from the study region of Augsburg. Altogether 1532 subjects (755 men, 777 women; 614 index probands, 918 full sibs and parents) aged 20 to 98 years could be examined in the KORA (Cooperative health research in the region of Augsburg) study centre. For the present study only index probands with known type 2 diabetes were included (n = 614). We excluded from the present analysis 33 subjects with missing values on any of the considered risk factors. Finally, the analyses comprised 581 type 2 diabetic subjects (352 men and 229 women) aged 29 to 83 years.

The study was conducted according to the principles expressed in the Declaration of Helsinki as revised in 2000 and was approved by the ethics committee of the Bavarian Medical Association. All participants gave their written informed consent.

### Data collection

Baseline information on sociodemographic variables, smoking habits, physical activity level, medication use, parental history of diseases, and alcohol consumption were gathered by trained medical staff (mainly nurses) during a standardized face to face interview. Individuals with known type 2 diabetes were asked to report their age at disease onset. Anthropometric measurements were made by trained personnel, subjects wearing light indoor clothing and no shoes. Body weight was measured to the nearest 0.1 kg, height to the nearest mm, and waist and hip circumferences to the nearest mm while the proband was standing. Blood pressure was measured at the right arm in a sitting position after a fifteen-minute rest using a validated automatic device (OMRON 705-CP); 3 measurements were taken with 3-minute intervals between the measurements. For the present analysis, the results of the second and third measurements were averaged

### Laboratory procedures

Total cholesterol was determined by cholesterol-esterase method (CHOL Flex, Dade-Behring, Germany), HDL cholesterol using the AHDL Flex (Dade-Behring), and LDL cholesterol was measured by a direct homogeneous method (GreinerBiochemica, Germany), respectively. Urinary albumin and creatinine levels were determined in a random spot urine specimen (Tina-Quant, Roche Diagnostics for the measurement of urinary albumin and KREA Flex, Dade-Behring, for the measurement of urinary creatinine). Serum creatinine was determined using a modified Jaffe test (KREA Flex, Dade-Behring), and serum uric acid was measured using an enzymatic colorimetric test (URCA Flex, Dade-Behring). HbA1c was determined by a turbidimetric immunological inhibition assay (TINIA, Dade-Behring).

### Definition of variables

Body mass index (BMI) was calculated as weight in kilograms divided by height in square meters and waist-to-hip ratio as waist divided by hip circumference. Dyslipidemia was defined as total cholesterol to HDL cholesterol ratio ≥ 5. Education level was categorized into 'low' (< 12 years of schooling) and 'high' (≥ 12 years of schooling). A regular smoker was defined as a subject who smoked at least one cigarette per day. Alcohol intake was classified into the three categories 0, 0.1–19.9, and 20.0 or more g/day for women and 0, 0.1–39.9, and 40 or more g/day for men. Participants were defined as active during leisure time if they regularly participated in sports in summer and winter and if they were active for at least 1 hour per week in either season. The abbreviated Modification of Diet in Renal Disease (MDRD) Study Group equation was used to calculate eGFR: eGFR (mL/min/1.73 m^2^) = 186.3 × (serum creatinine^-1.154^) × (age^-0.203^) × 0.742 (if female) × 1.212 (if black) [11]. Microalbuminuria was defined as an albumin-creatinine ratio of 30 to 300 mg/g, and macroalbuminuria as an albumin-creatinine ratio of more than 300 mg/g.

Self-reported type 2 diabetes was validated by hospital records or by contacting the proband's treating physician.

### Target definitions

To be comparable with previous studies, due to the guidelines from the European Society of Hypertension-European Society of Cardiology [12], the American Diabetes Association [13], and the Joint National Committee VII [14], the following recommended targets were considered in the present paper: < 7.0% for HbA1c, < 130/85 mmHg for blood pressure, < 100 mg/dl for LDL cholesterol, < 200 mg/dl for total cholesterol, < 25 kg/m^2 ^for BMI, and > 45 mg/dl (men) and > 55 mg/dl (women) for HDL cholesterol.

### Statistical analysis

Means or proportions for baseline clinical and laboratory characteristics were computed for subjects with and without micro- or macroalbuminuria. The Chi^2^-Test was used to test the differences in prevalences. The general linear model was used to compare means (F-Test). Multiple logistic regression analysis was done using albuminuria (including micro- and macroalbuminuria) as the dependent variable and age, sex, BMI, waist circumference, diastolic blood pressure, systolic blood pressure, total cholesterol values, HbA1c values, duration of diabetes, serum creatinine, and regular smoking as independent variables. Significance tests were two tailed and p-values less than 0.05 are stated as statistically significant. All analyses were performed using the Statistical Analysis System (Version 9.1, SAS Institute Inc., Cary, NC).

## Results

Microalbuminuria was revealed in 158 of 581 diabetic persons (27.2%) and proteinuria was found in 52 of 581 diabetic persons (9.0%).

### Characteristics of the study participants

The clinical and laboratory characteristics of all participants included in the study and of the subjects with and without micro- or macroalbuminuria are shown in Table 1. Subjects with microalbuminuria or proteinuria had a significantly higher WHR, a higher waist circumference, higher HbA1c values, higher creatinine values, higher total cholesterol as well as LDL cholesterol values, higher uric acid levels, and higher systolic and diastolic blood pressure values than subjects with normoalbuminuria. Diabetic persons with microalbuminuria or proteinuria were more frequently of male gender, and the duration of diabetes was significantly longer compared to normoalbuminuric subjects. The GFR values were significantly lower than in those without microalbuminuria or proteinuria. There were no significant differences with regard to BMI, regular smoking, physical activity, alcohol consumption, and history of CVD between subjects with microalbuminuria or proteinuria and normoalbuminuric participants.

**Table 1:** Clinical characteristics and laboratory parameters in all study participants and diabetic participants with normoalbuminuria, microalbuminuria, and macroalbuminuria

**Characteristics**	**All study participants**	**Normo-albuminuric**	**Micro-albuminuric**	**Macro-albuminuric**	P***
N	*N = 581*	*n = 371*	*n = 158*	*n = 52*	
					
Age (years)	61.0 (9.5)	60.3 (9.9)	62.3 (8.8)	61.7 (8.5)	0.0859
Men (%)	60.6	56.3	63.3	82.7	0.0009
Duration of diabetes (years)	9.5 (8.3)	8.3 (7.5)	11.3 (9.1)	13.2 (9.4)	< 0.0001
BMI (kg/m^2^)	30.8 (5.3)	30.6 (5.2)	31.0 (5.3)	31.1 (6.4)	0.6640
Waist-hip ratio	0.95 (0.08)	0.94 (0.07)	0.96 (0.08)	0.97 (0.08)	0.0005
Waist circumference (cm)	105.2 (12.8)	104.1 (12.4)	106.8 (13.4)	108.5 (13.0)	0.0129
Systolic blood pressure (mmHg)	140.0 (21.7)	135.2 (19.4)	145.6 (21.1)	157.7 (26.0)	< 0.0001
Diastolic blood pressure (mmHg)	80.8 (10.9)	79.9 (10.5)	82.0 (11.4)	83.7 (11.5)	0.0158
Regular smoker (%)	13.9	12.4	15.2	21.2	0.2023
Physical inactivity (%)	57.3	56.6	57.0	63.5	0.6415
Alcohol intake 0 g/day (%)	41.3	40.7	39.9	50.0	0.4380
0.1–39.9 (men) 0.1–19.9 (women) (%)	45.3	46.1	47.5	32.7	
≥ 40 g/day (men) ≥ 20 g/day (women) (%)	13.4	13.2	12.7	17.3	
History of cardiovascular events (%)	27.7	25.3	29.8	38.5	0.1124
HbA1c (%)	7.3 (1.4)	7.1 (1.2)	7.5 (1.6)	8.1 (1.8)	< 0.0001
Serum creatinine (mg/dl)*	1.04 (1.33)	0.99 (1.24)	1.09 (1.43)	1.26 (1.47)	< 0.0001
GFR (ml/min per 1.73 m^2^)**	71.6 (18.9)	73.7 (16.9)	69.5 (21.0)	63.2 (22.2)	0.0002
Total cholesterol (mg/dl)	207.4 (42.2)	203.7 (39.6)	212.5 (44.7)	218.2 (49.9)	0.0139
LDL cholesterol (mg/dl)	129.0 (30.7)	126.3 (28.2)	132.7 (32.8)	137.2 (37.9)	0.0109
HDL cholesterol (mg/dl)	50.0 (14.8)	50.5 (13.9)	49.3 (17.1)	48.4 (13.5)	0.5150
Serum uric acid (mg/dl)	5.7 (1.6)	5.5 (1.4)	6.0 (1.7)	6.3 (1.9)	< 0.0001

Data are means (± SD) unless otherwise indicated. Cardiovascular events include acute myocardial infarction or stroke

* geometric mean

**estimated from the MDRD equation

***p-value for differences between type 2 diabetes subjects with normo-, micro- and macroalbuminuria

### Risk factors associated with albuminuria

In the multiple logistic regression analysis using albuminuria as the dependent variable a number of risk factors associated with albuminuria could be identified. It was found that systolic blood pressure, HbA1c, duration of diabetes, serum creatinine values, regular smoking and waist circumference were significantly associated with albuminuria (Table 2).

**Table 2:** Multiple logistic regression analysis using albuminuria as a dependent variable

**Variable**	**OR (95% CI)**
HbA1c (%)	1.25 (1.09 – 1.44)
Duration of diabetes (years)	1.05 (1.02 – 1.07)
Systolic blood pressure (mmHg)	1.03 (1.02 – 1.04)
Serum creatinine (mg/dl)*	4.27 (2.09 – 8.70)
Regular smoking	2.07 (1.20 – 3.57)
Waist circumference (cm)	1.02 (1.01 – 1.04)

*Serum creatinine log-transformed

### Antidiabetic and cardiovascular drug treatment

Table 3 presents the antidiabetic treatment as well as the treatment with antihypertensive/cardiovascular drugs in the different groups. Normoalbuminuric subjects were more often treated with oral antidiabetic medication only (42.1%) in comparison to microalbuminuric (30.4%) and macroalbuminuric (30.8%) persons. Contrary, subjects with micro- and macroalbuminuria were significantly more often treated with insulin only (38.6% and 36.5%, respectively) or insulin in combination with oral antihyperglycemics (26.0% and 30.8%, respectively) than normoalbuminuric persons.

**Table 3:** Antidiabetic medication in diabetic participants with normoalbuminuria, microalbuminuria, and macroalbuminuria

**Medication**	**Normoalbuminuric**	**Microalbuminuric**	**Macroalbuminuric**	**P**
N	*n = 371*	*n = 158*	*n = 52*	
				
***Antidiabetic treatment***				
*Oral antidiabetic medication only (%)*	42.1	30.4	30.8	0.0062
*Insulin only (%)*	26.7	38.6	36.5	
*Oral antidiabetic medication in combination with insulin (%)*	20.2	26.0	30.8	
*No antidiabetic drug*	11.1	5.1	1.9	
***Antihypertensive/Cardiovascular medication***				
*Beta blockers (%)*	40.2	38.6	57.7	0.0401
*ACE inhibitors (%)*	44.7	46.2	48.1	0.8801
*Diuretic use (%)*	37.5	50.0	51.9	0.0092
*AT*_*1 *_*blockers (%)*	8.9	9.5	15.4	0.3311
*Calcium channels blockers (%)*	17.8	22.8	23.1	0.3353
*Lipid lowering drugs (%)*	37.2	36.7	42.3	0.7523
*Antiplatelet drugs (%)*	44.7	45.6	44.2	0.9794

In comparison with normoalbuminuric participants, subjects with micro- and macroalbuminuria were significantly more frequently treated with diuretics (37.5%, 50.0% and 51.9%, respectively) and beta-blockers (40.2%, 38.6%, and 57.7%, respectively). For ACE inhibitors, AT_1 _blockers, calcium channel blockers as well as lipid lowering drugs and antiplatelets there were no statistically significant differences between the three groups (Table 3).

### Glycemic control and treatment of cardiovascular risk factors

In this study only a small proportion of persons with type 2 diabetes reached adequate glycemic control as well as optimal treatment of modifiable cardiovascular risk factors according to guidelines. HbA1c value was < 7% in 46.6%, total cholesterol was < 200 mg/dl in 44.1%, and LDL cholesterol was < 100 mg/dl in 16.0% of the participants. Optimal HDL cholesterol values were found in 55.8%, and blood pressure values < 130 and < 85 mmHg in 31.3% of the persons (Table 4).

**Table 4:** The percentage of type 2 diabetic participants who achieved optimal treatment goals for modifiable risk factors

**Risk factors**	**%**
HbA1c < 7%	46.6
Total cholesterol < 200 mg/dl	44.1
HDL cholesterol > 45 mg/dl (men), > 55 mg/dl (women)	55.8
LDL cholesterol < 100 mg/dl	16.0
Blood pressure < 130 and 85 mmHg	31.3
BMI < 25 kg/m^2^	9.8
Current non-smoker	86.1

## Discussion

The present study conducted in a study sample of type 2 diabetic persons recruited from the general population with median diabetes duration of 7.0 years showed that microalbuminuria (27.2%) and macroalbuminuria (9.0%) is common among German persons with known type 2 diabetes. Logistic regression modeling identified HBA1c, duration of diabetes, systolic blood pressure, serum creatinine, smoking and waist circumference as independent risk factors associated with albuminuria. In this study neither adequate glycemic control nor optimal treatment of modifiable cardiovascular risk factors according to guidelines was achieved in persons with type 2 diabetes.

Because the results of hitherto existing studies on this issue are based on different population characteristics (ethnicity, duration of diabetes, age-range, inclusion of type 1 and type 2 diabetic persons) direct comparisons with regard to the prevalence of microalbuminuria and macroalbuminuria between the investigations are difficult. So far, only a few studies of albuminuria in patients with type 2 diabetes anywhere in the world were population-based [15-18]. In the Third National Health and Nutrition Examination Survey, the prevalence of albuminuria was 34% (28.1% microalbuminuria, 6.1% macroalbuminuria) among persons with previously diagnosed type1 and type 2 diabetes [17], whereas in a study from Italy [16] the prevalence of albuminuria was 49.7% in known type 2 diabetic persons (32.1% microalbuminuria, 17.6% macroalbuminuria). In a prior German study including patients with recently diagnosed type 2 diabetes 19% exhibited microalbuminuria and 5.2% macroalbuminuria [19]. Furthermore, another study including diabetic patients treated in primary care reported a prevalence of microalbuminuria (macroalbuminuria) of 17.2% (10.8%) in Type 2 diabetes [20]. Although the present study was not population-representative the proportion of microalbuminuria and macroalbuminuria is similar to that reported in other studies and gives therefore a realistic estimation of the prevalence of albuminuria among type 2 diabetic persons in Germany [16-20].

In the present study HbA1c level, duration of diabetes, systolic blood pressure, serum creatinine, regular smoking, and waist circumference were significantly associated with albuminuria. This finding is in agreement with other studies, which also reported that diabetes duration [18,21], and hypertension [18,21] are important factors in the development of albuminuria. So far, only a few studies have found that smoking is a risk factor for albuminuria among persons with type 2 diabetes [22]. In this study, persons who smoked regularly were twice as likely to have albuminuria compared with those who did not. Furthermore, in agreement with other studies, in our study HbA1c levels were also associated with the development of albuminuria [23]. While other studies mostly identified BMI as risk factor for albuminuria [18], in our study waist circumference was more strongly associated with albuminuria than BMI.

In the present study persons with micro- or macroalbuminuria were more frequently treated with insulin alone or with insulin in combination with oral antidiabetic drugs in comparison to normoalbuminuric type 2 diabetes mellitus persons. This result is plausible and in agreement with findings from other studies [24], because intensification of treatment reflects the natural progression of the disease; due to long-standing disease complications and comorbidities occur which require insulin therapy to manage type 2 diabetes persons.

Results from randomized clinical trials demonstrated that control of glucose levels as well as blood pressure, and cholesterol levels can delay or prevent the macrovascular and microvascular complications of diabetes [25-28]. Despite the guidelines, developed on the basis of such data, in the present study only a small proportion of persons with type 2 diabetes achieved the recommended levels of control. This is in accordance with results from other studies [24,29-31].

The Augsburg Diabetes Family Study has several limitations that need to be considered. The present study was cross-sectional in design, thus, longitudinal trends in the management of diabetes and modifiable risk factors could not be taken into account. Furthermore, a single random spot urine sample was used to determine albuminuria. Unfortunately, in the present study no data on the reproducibility of the urinary albumin excretion measurement was available. Although the study participants were recruited from the general population the present study was not population representative in design. Furthermore, selection bias cannot be excluded, as the study population could be expected to include an above average proportion of motivated persons with type 2 diabetes. This selection may have underestimated the reported prevalences of albuminuria and shortcomings in disease management.

However, the present study has also a number of strengths. Contrary to some prior studies, which were conducted in selected, e.g. clinic-based, patient groups [21,31] the present study was based on study participants recruited from the general population. Also, studies on this issue based on a well characterized study population as the present one including a great number of phenotypes, risk factors and comorbidities are scarce. Furthermore, contrary to other papers on this subject [17,18] the present paper additionally contains data on antidiabetic and cardiovascular treatment. In addition, this study is one of relatively few from Germany dealing with microalbuminuria. Thus, since type 2 diabetes is one of the most common health problems in primary care, we believe that our results can be applied to improve the management of cardiovascular risk factors, glycemic control and microalbuminuria in patients with type 2 diabetes.

## Conclusion

In conclusion, the present study could show that the prevalences of microalbuminuria and macroalbuminuria in a German sample of persons with type 2 diabetes were comparable to those shown in other studies. Despite the high risk of cardiovascular events and mortality among type 2 diabetic persons, in our study control of diabetes and risk factors was not optimal. Thus, glycemic control and management of cardiovascular risk factors should be intensified to effectively reduce the risk of micro- and macrovascular complications in diabetic persons.

## Competing interests

The authors declare that they have no competing interests.

## Authors' contributions

CM contributed to the conception of the paper, analysed and interpreted the data and drafted the manuscript. MH, RL, MH, HEW and WP contributed to the conception of the paper and the interpretation of the data. All authors read and approved the final manuscript.

## Pre-publication history

The pre-publication history for this paper can be accessed here:


